# Unexpected Reaction Pathway for butyrylcholinesterase-catalyzed inactivation of “hunger hormone” ghrelin

**DOI:** 10.1038/srep22322

**Published:** 2016-02-29

**Authors:** Jianzhuang Yao, Yaxia Yuan, Fang Zheng, Chang-Guo Zhan

**Affiliations:** 1 Molecular Modeling and Biopharmaceutical Center and College of Pharmacy, University of Kentucky, 789 South Limestone Street, Lexington, KY 40536, USA; 2 Department of Pharmaceutical Sciences, College of Pharmacy, University of Kentucky, 789 South Limestone Street, Lexington, KY 40536, USA

## Abstract

Extensive computational modeling and simulations have been carried out, in the present study, to uncover the fundamental reaction pathway for butyrylcholinesterase (BChE)-catalyzed hydrolysis of ghrelin, demonstrating that the acylation process of BChE-catalyzed hydrolysis of ghrelin follows an unprecedented single-step reaction pathway and the single-step acylation process is rate-determining. The free energy barrier (18.8 kcal/mol) calculated for the rate-determining step is reasonably close to the experimentally-derived free energy barrier (~19.4 kcal/mol), suggesting that the obtained mechanistic insights are reasonable. The single-step reaction pathway for the acylation is remarkably different from the well-known two-step acylation reaction pathway for numerous ester hydrolysis reactions catalyzed by a serine esterase. This is the first time demonstrating that a single-step reaction pathway is possible for an ester hydrolysis reaction catalyzed by a serine esterase and, therefore, one no longer can simply assume that the acylation process must follow the well-known two-step reaction pathway.

Obesity is well known as a major public health problem, and it is related to the worldwide increasing rates of the disease and burden of the associated co-morbidities, such as type 2 diabetes, cardiovascular disease, and cancer^1^. More than one third of adult population in the world are reported to be in the risk of overweight-caused diseases, *e.g.* diabetes mellitus, hypertension, coronary heart disease, stroke, gall bladder disease, osteoarthritis, and dyslipidaemia^2^,^3^,^4^. It is crucial for improving health of people in the risk of overweight-caused diseases to lose weight. Unfortunately, caloric restriction (*e.g*. fasting) is commonly accompanied by elevated appetite^5^, which makes weight loss a great challenge for obese individuals. In fact, more than 30 billion dollars were spent annually in the United States on reduced-calorie and multitudinous diets^6^. However, such diets did not show significant efficacy in weight loss^7^.

An effective pharmacological treatment is needed to counteract the increased appetite during fasting. However, FDA-approved pharmaceutical agents for obesity treatment are accompanied by serious adverse/toxic effects and, thus, a number of pharmaceutical agents (*e.g*. aminorex, dexfenfluramine, fenfluramine, and phenylpropanolamine) have been withdrawn from the market^8^,^9^. Currently available FDA-approved anti-obesity drugs include phentermine, lorcaserin, orlistat, and phentermine/topiramate ER, still with a variety of adverse side effects, such as insomnia, headache, diarrhea, dizziness, depression, and many others^10^. It is highly desired to develop a new, safe, and truly effective pharmacological agent for obesity treatment.

Interestingly, a gastric peptide hormone, known as ghrelin or the “hunger hormone”, was discovered in 1999 by Kojima *et al.*^11^. Ghrelin is a 28-amino acid peptide (GSSFLSPEHQKAQQRKESKKPPAKLQPR) with Ser3 side chain acylated by a fatty acid (*n*-octanoic acid), as shown in Fig. 1A. So, ghrelin has an *n*-octanoylated peptide. The 28-amino acid peptide without acylation (*n*-octanoylation) is known as desacyl-ghrelin^12^. This acylation, *via* ghrelin O-acyltransferase enzyme (GOAT), is essential for the activity of ghrelin with growth hormone secretagogue receptors (GHSRs) in the central nervous system (CNS) that mediate hyperphagia and adiposity^13^. Ghrelin was identified in human stomach. It is believed that ghrelin is released primarily from cells in the stomach and travels to the brain through blood circulation. In the brain, ghrelin interacts with both hypothalamus (the brain’s physiological eating center) and the brain’s pleasure centers to arouse hunger^14^. In fact, ghrelin is the only known hormone stimulating hunger and food intake^1^, telling you when to eat. Ghrelin level naturally changes dramatically during the course of a day. Ghrelin level rises steeply with fasting (or before a meal) and decreases after a meal^14^. Ghrelin is emerging as a novel, potentially attractive anti-obesity drug target^8^.

Recent drug discovery efforts have been centralized on ghrelin and aim to reduce the appetite of overweight people by different approaches, including regulation of ghrelin release, ghrelin receptor antagonism, and reducing active ghrelin production by inhibition of GOAT^15^,^16^,^17^,^18^,^19^. However, there is still no clinically useful drug identified so far.

Based on the aforementioned physiological and biological mechanisms of ghrelin, an ideal therapeutic approach would directly inactivate ghrelin itself and convert ghrelin to its inactive form (desacyl-ghrelin) by using an efficient ghrelin-metabolizing enzyme (if available). For example, inhibition of GOAT would decrease the production of (active) ghrelin, but cannot inactivate ghrelin that has already generated in the body. A ghrelin-metabolizing enzyme would directly inactivate ghrelin by converting ghrelin to desacyl-ghrelin and, thus, more effectively decrease the appetite. Despite of extensive effort that aims to regulate the ghrelin level in the body, there is no report of a therapeutic candidate which can be used to directly inactivate ghrelin by converting ghrelin to desacyl-ghrelin.

More recently reported studies have shed light on the possibility of ghrelin hydrolysis in human butyrylcholinesterase (BChE). It was first reported that ghrelin can be hydrolyzed to the inactive form, *i.e*. desacyl-ghrelin, by BChE^20^, but the enzyme activity was not characterized, without knowing the kinetic parameters for BChE-catalyzed ghrelin hydrolysis. Lockridge *et al.*^21^ demonstrated that the BChE knockout mice became overweight on a high-fat diet compared to the control mice, suggesting that BChE is essential for ghrelin inactivation. Most recently reported studies^22^,^23^ provided more convincing evidence for the role of human BChE in hydrolyzing ghrelin to desacyl-ghrelin, and the catalytic parameters for BChE-catalyzed hydrolysis of ghrelin were estimated (*k*_cat_ = ~1.4 min^−1^ and *K*_M_ = ~1 μM)^22^. Obviously, with *k*_cat_ = ~1.4 min^−1^, the catalytic activity of wild-type BChE against ghrelin is rather low. It is highly desired for development of a novel, safe and effective anti-obesity drug to design a mutant of human BChE with a significantly improved catalytic activity against ghrelin.

Here we propose, for the first time, that a BChE mutant with significantly improved catalytic activity against ghrelin could be used as an exogenous enzyme to control appetite and for anti-obesity treatment. Human BChE is an ideal protein scaffold to develop a therapeutic enzyme, because it has been proven safe for use as an exogenous enzyme in humans, and it has the highly desired thermal stability of a therapeutic protein. In a relevant effort, starting from computational study of the reaction pathway for BChE-catalyzed hydrolysis of cocaine^24^, we have successfully designed and discovered BChE mutants with considerably improved catalytic activity against cocaine for the purpose of developing an enzyme therapy for treatment of cocaine abuse^25^,^26^,^27^,^28^,^29^,^30^,^31^,^32^. In fact, one of our designed and discovered BChE mutants is currently under Phase II clinical trial for cocaine addiction treatment by Teva Pharmaceuticals^33^,^34^,^35^. The similar computational strategy used to develop an anti-cocaine enzyme therapy may be used to develop an anti-obesity enzyme therapy.

For rational design of a BChE mutant with improved catalytic activity against ghrelin, one must first uncover and understand the detailed reaction pathway and the corresponding free energy profile for BChE-catalyzed hydrolysis of ghrelin, as we did for BChE-catalyzed hydrolysis of cocaine^24^. So, as the first step of a long-term effort towards rational design and discovery of a highly active BChE mutant against ghrelin, this computational study aimed to uncover the fundamental reaction pathway for BChE-catalyzed hydrolysis of ghrelin by perform quantum mechanical/molecular mechanical (QM/MM) reaction-coordinate calculations, molecular dynamics (MD) and free energy simulations. Based on the calculations and simulations, BChE-catalyzed hydrolysis of ghrelin follows a unique reaction pathway which is remarkably different from the usual reaction pathway known for all ester hydrolysis reactions catalyzed by an esterase such as acetylcholinesterase (AChE), BChE, or any other serine esterase. As well-known, an enzymatic ester hydrolysis consists of acylation and deacylation processes (as shown in Fig. 1B,C). According to the well-known usual reaction pathway, the acylation stage of the ester hydrolysis is a two-step process with a tetrahedral intermediate between two transition states. Surprisingly, for BChE-catalyzed hydrolysis of ghrelin, the acylation is a single-step process according to our calculations and simulations, and this single-step acylation process is rate-determining. The free energy barrier calculated for the rate-determining step is in good agreement with available experimental kinetic data. The novel mechanistic insights obtained from this computational study should be valuable not only in guiding future rational design and discovery of BChE mutants with improved catalytic activity against ghrelin, but also in future studies on catalytic mechanisms of many other enzymatic ester hydrolysis reactions as it has shown that a single-step acylation pathway is possible.

## Results

### BChE-ghrelin binding mode

The MD simulations at the QM/MM(SCC-DFTB:CHARMM27) level, in which the QM calculation was performed at the self-consistent charge density functional tight-binding (SCC-DFTB) level^36^ while the MM calculation was performed using the CHARMM27 force field^37^, revealed that the first four residues of ghrelin occupy the active site of human BChE very well. The key catalytic residues of BChE include the catalytic triad (consisting of Ser198, His438, and Glu325) and oxyanion hole (consisting of the backbone NH groups of Gly116, Gly117, and Ala199). According to the simulated structure (see Figure S1 in Supporting Information), the hydroxyl oxygen on the Ser198 side chain of BChE aligns well with the carbonyl carbon on the *n*-octanoylated Ser3 side chain of ghrelin, ready for nucleophilic attack. Meanwhile, the carbonyl oxygen of the *n*-octanoylated Ser3 side chain of ghrelin stays in the oxyanion hole, forming hydrogen bonds with the backbone NH groups of Gly117 and Ala199. In addition to the catalytic residues, N-terminal ammonium group of ghrelin forms a cation-π interaction with Trp82 of BChE and a salt bridge with Asp197 side chain. The hydroxyl group on the side chain of Ser2 of ghrelin forms a hydrogen bond with the oxygen atom of His438 backbone. The Phe4 side chain of ghrelin locates in a hydrophobic cave formed by Ala328, Phe329, Tyr332, and Tyr430. These favorable interactions help to stabilize ghrelin binding in the active site of BChE.

### Acylation reaction pathway

**Figure 1:**
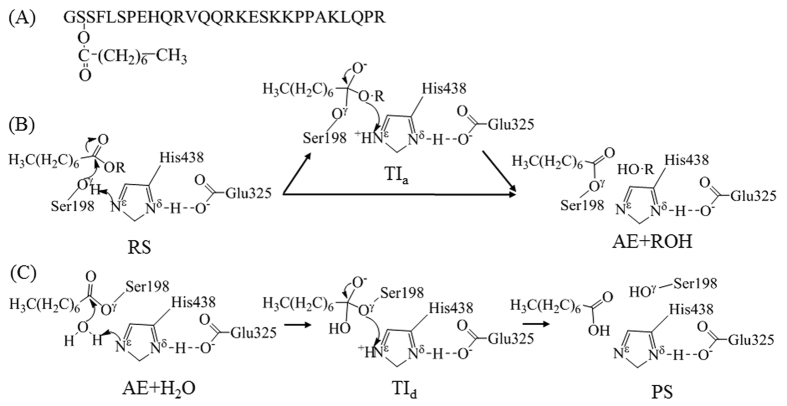
Possible reaction pathways for human BChE-catalyzed hydrolysis of ghrelin. (**A**) Amino acid sequence of human ghrelin. (**B**) Acylation process (with or without existence of a tetrahedral intermediate) in which ghrelin is represented as CH_3_(CH_2_)_6_COOR: Reactant State (RS), Tetrahedral Intermediate (TI_a_), and Acyl-Enzyme (AE) + desacyl-ghrelin (ROH). (C) Deacylation process: Acyl-Enzyme (AE) + H_2_O, Tetrahedral Intermediate (TI_d_), and Product State (PS).

**Figure 2:**
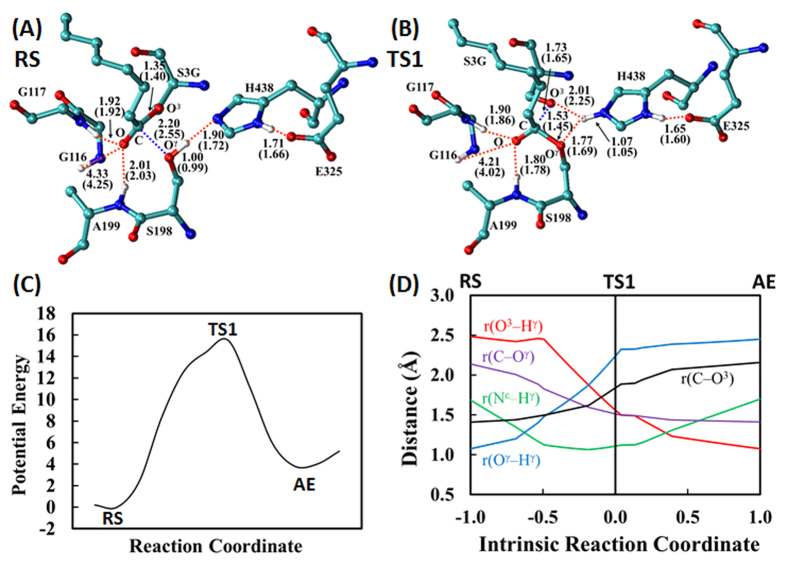
Optimized geometries of (**A**) the reactant state (RS) and (**B**) the transition state (TS1) for the acylation process of BChE-catalyzed hydrolysis of ghrelin. Indicated in the figures are the internuclear distances (Å) optimized at the QM/MM(SCC-DFTB:CHARMM27) level, and the values in parentheses refer to the distances optimized at the QM/MM(B3LYP/6-31G*:CHARMM27) level. (**C**) Plot of the potential energy *vs* the reaction coordinate (RC1) used the in the QM/MM(SCC-DFTB:CHARMM27) reaction-coordinate calculations; RC1 = r(C−O^3^) – r(C−O^γ^). (**D**) Plots of key internuclear distances *vs* the intrinsic reaction coordinate (IRC) calculations at the QM/MM(SCC-DFTB:CHARMM27) level. The distances shown include the following atoms: C (carbonyl carbon atom on the *n*-octanoylated Ser3 side chain of ghrelin); O^3^ (ester oxygen atom on the *n*-octanoylated Ser3 side chain of ghrelin); O^γ^ (hydroxyl oxygen atom on Ser198 side chain of BChE); H^γ^ (hydroxyl hydrogen on the *n*-octanoylated Ser3 side chain of ghrelin); and N^ε^ (nitrogen atom on His438 side chain of BChE).

Starting from the BChE-ghrelin binding structure (Michaelis-Menten complex) as the reactant state (RS depicted in Fig. 1), we performed the QM/MM reaction-coordinate calculations at both the QM/MM(SCC-DFTB:CHARMM27) and QM/MM(B3LYP/6-31G*:CHARMM27) levels in which the QM calculation was performed at the SCC-DFTB or B3LYP/6-31G* level while the MM calculation was carried out using the CHARMM27 force field. In addition, all geometries optimized at the QM/MM(B3LYP/6-31G*:CHARMM27) level were used to perform further single-point energy calculations at the QM/MM(B3LYP/6-311++G**:CHARMM27) level. The QM/MM calculations using different reaction coordinates at various QM/MM levels consistently revealed that the acylation of human BChE with ghrelin is a single-step reaction process, as shown in Fig. 2 (and figures in Supporting Information). The acylation process is mainly reflected by the formation of a new covalent bond (C−O^γ^) between the carbonyl carbon (C) on the *n*-octanoylated Ser3 side chain of ghrelin and the hydroxyl oxygen (O^γ^) on Ser198 side chain of BChE and breaking of a covalent bond (C−O^3^) between the carbonyl carbon (C) and the ester oxygen (O^3^) on the *n*-octanoylated Ser3 side chain of ghrelin. Hence, a linear combination of these two bond lengths, r(C−O^3^) – r(C−O^γ^), was used as the reaction coordinate (RC), denoted as RC1 for convenience, for the QM/MM reaction-coordinate calculations. In further intrinsic reaction coordinate (IRC) calculations at the QM/MM(SCC-DFTB:CHARMM27) level starting from the optimized transition state (TS1) structure, TS1 smoothly went to the acyl-enzyme (AE) in the forward direction and the reactant state (RS) in the backward direction, which supports that there is only one transition state between RS and AE.

According to the QM/MM reaction-coordinate calculations, starting from the RS (Fig. 2A), the acylation process involves only one transition state (TS1 depicted in Fig. 2B): the nucleophilic attack proceeds as the hydroxyl oxygen (O^γ^) of Ser198 of BChE gradually approaches the carbonyl carbon (C) on the *n*-octanoylated Ser3 side chain of ghrelin, while the ester oxygen (O^3^) atom of the *n*-octanoylated Ser3 side chain gradually leaves the carbonyl carbon. Meanwhile, the hydroxyl hydrogen (H^γ^) of the *n*-octanoylated Ser3 side chain gradually leaves O^γ^ and approaches the ester oxygen (O^3^) atom of the *n*-octanoylated Ser3 side chain, as seen in Fig. 2D. As seen in Fig. 2D, while the O^3^–H^γ^ distance, denoted as r(O^3^–H^γ^), becomes shorter and shorter, the distance between H^γ^ and the nitrogen (N^ε^) atom on His438 side chain of BChE, denoted as r(N^ε^–H^γ^), becomes even shorter initially prior to reaching TS1 and then becomes longer and longer. The shortest N^ε^–H^γ^ distance is ~1.06 Å which is slightly longer than a typical N–H bond length. While r(N^ε^–H^γ^) = ~1.06 Å, r(C–O^3^) = ~1.62 Å and r(C–O^γ^) = ~1.60 Å; these C–O bond lengths are all significantly longer than a typical C–O bond length. So, it would be reasonable to say that H^γ^ first transfers to N^ε^ (but without a truly stable tetrahedral intermediate associated with a local minimum on the potential energy surface) and then transfers to O^3^. As a result, there is only one transition state, TS1, in which the C–O^3 ^bond has been partially broken, while the O^3^–H^γ^ bond has not yet been formed, as seen in Fig. 2B,D. So, it is also reasonable to say that the nucleophilic attack and the ester oxygen leaving are a concerted process (see Fig. 2C) which produces the acylated enzyme or acyl-enzyme (AE): *n*-octanoylated BChE. This concerted single-step acylation process is remarkably different from the well-known two-step acylation pathway (associated with an assumed tetrahedral intermediate, like TI_a_ indicated in Fig. 1, sandwiched by two transition states) known for numerous enzymatic ester hydrolysis reactions. For comparison and validation, we also tested the QM/MM reaction-coordinate calculations using an alternative RC (see Supporting Information), leading to the same conclusion that the assumed two-step pathway does not exist for BChE-catalyzed hydrolysis of ghrelin because the assumed tetrahedral intermediate is unstable.

According to this novel reaction pathway, in going from RS to TS1, the O^…^H distances involved in the hydrogen bonds between the oxyanion hole and carbonyl oxygen on the *n*-octanoylated Ser3 side chain of ghrelin all become significantly shorter in TS1, as seen in Fig. 2A,B. The enhanced hydrogen bonding helps to stabilize transition state TS1. Notably, there is no proton transfer between His438 and Glu325 within the catalytic triad during the acylation process.

In order to further validate the SCC-DFTB and B3LYP based QM/MM results, additional QM/MM reaction-coordinate calculations were performed on the acylation process by using the more recently developed B97-3 functional^38^ or ωM06-D3 functional^39^, instead of the B3LYP functional in the QM/MM calculations. Depicted in Figure 6S are the potential energy surfaces obtained from the QM/MM reaction-coordinate calculations at the QM/MM(B97-3/6-31G* :CHARMM27) and QM/MM(ωM06-D3/6-31G*:CHARMM27) levels and the corresponding single-point energy calculations at the QM/MM(B97-3/6-311++G**:CHARMM27) and QM/MM(ωM06-D3/6-311++G**:CHARMM27) levels. Comparing the data in Figure S6 with those in Figure S5, one can see that the three different DFT methods consistently led to the same conclusion that the acylation is a single-step reaction process. These additional QM/MM calculations support the reliability of the SCC-DFTB and B3LYP based QM/MM calculations.

Further, the combined QM/MM(SCC-DFTB:CHARMM27) MD and potential of mean force (PMF) simulations were carried out to simulate the acylation reaction process, accounting for the dynamics of the reaction process, and determine two-dimensional (2D) free energy maps associated with two different RC’s. The obtained 2D free energy maps (see Figure S2 in Supporting Information) for the acylation process demonstrate that the local minimum associated with pre-assumed tetrahedral intermediate TI_a_ indeed does not exist on the 2D free energy maps, and that there is only one transition state (TS1) existing between RS and AE. The 2D free energy maps provide further support to the finding that the acylation of human BChE with ghrelin is a single-step reaction process.

### Deacylation reaction pathway

**Figure 3:**
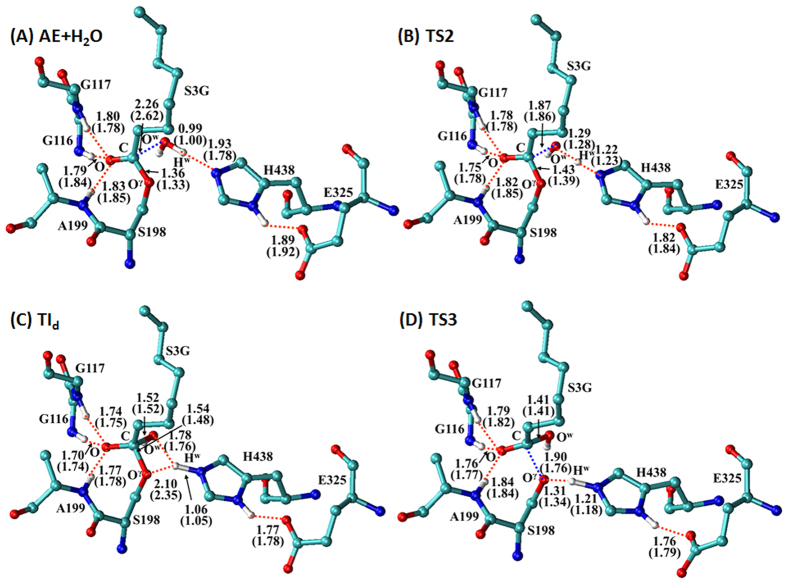
Optimized geometries of (**A**) the acyl-enzyme (AE) + H_2_O, (**B**) transition state TS2, (**C**) tetrahedral intermediate TI_d_, and (**D**) transition state TS3 during the deacylation process of BChE-catalyzed hydrolysis of ghrelin. Indicated in the geometries are the internuclear distances (Å) optimized at the QM/MM(SCC-DFTB:CHARMM27) level, and the corresponding values in parentheses refer to the distances optimized at the QM/MM(B3LYP/6-31G*:CHARMM27) level.

Starting from the AE structure, further reaction-coordinate calculations were carried out on the deacylation process at the same QM/MM levels used for the acylation process. The calculations using different reaction coordinates (RC’s) at all QM/MM levels consistently revealed that deacylation of AE follows a well-known two-step reaction pathway known for many other enzymatic ester hydrolysis reactions. As shown in Figs 1 and 3, during the chemical transformation from the acyl-enzyme (AE) to a tetrahedral intermediate (TI_d_) through transition state TS2, the oxygen (O^w^) atom of the nucleophilic water gradually approaches the carbonyl carbon (C) atom while a proton (H^w^) of the water molecule gradually transfers to the N_ε_ atom of His438 side chain. During the next reaction step from TI_d_ to the product state (PS depicted in Fig. 1), a proton gradually transfers from the nitrogen atom (N^ε^) atom of His438 side chain to the hydroxyl oxygen (O^γ^) atom of Ser198 side chain while the covalent bond C−O^γ^ gradually breaks. In practical QM/MM reaction-coordinate calculations leading to the results depicted in Fig. 3, the RC associated with TS2 was r(O^w^−H^w^) – r(C−O^w^) – r(N^ε^−H^w^), and the RC associated with TS3 was r(C−O^γ^) + r(N^ε^−H^w^) – r(O^γ^−H^w^). In addition, the QM/MM(SCC-DFTB:CHARMM27) reaction-coordinate calculations using a uniformed RC, *i.e.* RC4 = r(C−O^γ^) – r(C−O^w^), led to essentially the same results depicted in Fig. 3. As seen in Fig. 3, there is no proton transfer between His438 and Glu325 within the catalytic triad during the deacylation process.

In addition, the combined QM/MM(SCC-DFTB:CHARMM27) MD and PMF simulations were carried out to simulate the deacylation process, accounting for the dynamics of the reaction process, and determine 2D free energy maps associated with two different RC’s (Figure S3). The obtained 2D free energy maps are consistent with the finding that the deacylation process indeed follows the usual two-step reaction pathway known for numerous other enzymatic ester hydrolysis reactions.

### Overall free energy profile in comparison with available experimental data

**Figure 4:**
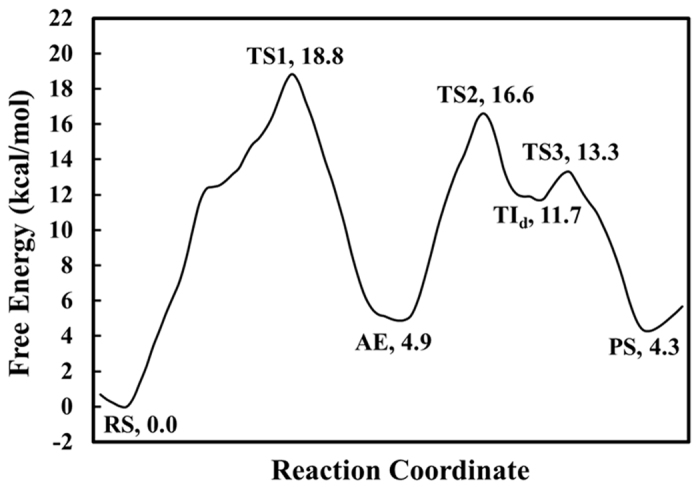
Minimum free energy profile determined by the QM/MM(SCC-DFTB:CHARMM27) calculations based two-dimensional PMF free energy maps for the entire reaction process (acylation and deacylation) of BChE-catalyzed hydrolysis of ghrelin.

Depicted in Fig. 4 is the minimum free energy profile plotted based on the 2D free energy maps (depicted in Figures S2 and S3). According to Fig. 4 and Figure S7, the entire BChE-catalyzed ghrelin hydrolysis process involves only three transition states (TS1 to 3), rather than the usually assumed four (or more) transition states known for enzymatic hydrolysis reactions of many other esters. No stable tetrahedral intermediate is found along the minimum free energy path of the acylation process. The free energy barriers associated with the three transition states (TS1 to 3) are 18.8 ± 0.1, 16.6 ± 0.5, and 13.3 ± 0.1 kcal/mol, respectively; the error bars were estimated based on the convergence of the PMF simulations with respect to the length of the production MD simulation run for each window (see Figure S7 in Supporting Information). Thus, the first reaction step (acylation) is rate-determining for BChE-catalyzed hydrolysis of ghrelin. The calculated highest free energy barrier of ~18.8 kcal/mol is reasonably close to the free energy barrier of ~19.4 kcal/mol derived from the available experimental rate constant (*k*_cat_ = 2.2 min^−1^)^38^ according to the conventional transition state theory^40^, suggesting that the obtained mechanistic insights are reasonable.

## Discussion

All of the QM/MM reaction-coordinate calculations, MD and PMF simulations have consistently demonstrated that the acylation process of BChE-catalyzed hydrolysis of ghrelin follows a single-step reaction pathway without existence of a stable tetrahedral intermediate between the reactant state (Michaelis-Menten complex) and acyl-enzyme, and that the deacylation is a two-step process. The single-step acylation process is rate-determining for the enzymatic hydrolysis of ghrelin. The free energy barrier of 18.8 kcal/mol calculated for the rate-determining step is reasonably close to the experimentally derived free energy barrier of ~19.4 kcal/mol, suggesting that the obtained mechanistic insights are reasonable.

The single-step reaction pathway for the acylation is remarkably different from the well-known two-step acylation reaction pathway for numerous ester hydrolysis reactions catalyzed by a serine esterase. It should be noted that the question of whether a tetrahedral intermediate exists at all has been a long discussion for uncatalyzed ester hydrolysis^41^,^42^. But the discussion has not involved an enzyme for catalysis. This is the first time demonstrating that a single-step reaction pathway is possible for an ester hydrolysis reaction catalyzed by a serine esterase and, therefore, one no longer can simply assume that the acylation process must follow the well-known two-step reaction pathway for the enzymatic ester hydrolysis.

In addition, the novel mechanistic insights and the rate-determining transition-state structure obtained in this computational study may be used in the future to guide rational design and discovery of a therapeutically valuable BChE mutant with improved catalytic efficiency against ghrelin. Generally speaking, for the purpose of future rational design of a BChE mutant with improved catalytic activity against ghrelin, it is important to understand why the free energy barrier (18.8 kcal/mol) calculated for the acylation process is significantly higher than those (16.6 and 13.3 kcal/mol) calculated for the deacylation process. One of the most crucial factors affecting the catalytic rate constant of a serine esterase is the hydrogen bonding between the carbonyl oxygen of the ester to be hydrolyzed and the oxyanion hole of the esterase. Such type of hydrogen bonding helps to stabilize the transition states and, thus, lower the free energy barriers, because the carbonyl oxygen will generally have more and more (partial) negative charge to make the hydrogen bonding stronger and stronger in going from the reactant state or acyl-enzyme to the transition state during the initial step of the acylation or deacylation step. In general, the overall hydrogen bonding with the carbonyl oxygen is expected to be stronger in the transition state compared to that in the reactant state or acyl-enzyme. Specifically for BChE-catalyzed hydrolysis of ghrelin, the carbonyl oxygen of the *n*-octanoylated Ser3 side chain has only two hydrogen bonds with the oxyanion hole of BChE (specifically with the NH groups of Gly117 and Ala199) during the acylation process, as seen in Fig. 2. The carbonyl oxygen has no hydrogen bond with Gly116 of the oxyanion hole. In comparison, the same carbonyl oxygen has three hydrogen bonds with all residues of the oxyanion hole, specifically with the NH groups of Gly116, Gly117, and A199 during the deacylation process. The difference between the acylation and deacylation in the hydrogen bonding is likely one of the possible main reasons for the relatively higher free energy barrier of the acylation process. Hence, the focus for further effort to design a BChE mutant with improved catalytic activity against ghrelin should aim to identify possible amino-acid mutations (particularly on the amino-acid residues nearby the oxyanion hole) that can enhance the overall hydrogen bonding of the carbonyl oxygen with the oxyanion hole, including an additional hydrogen bonding with the NH group of Gly116. The similar computational strategy has successfully been used in our previous studies^26^,^27^,^32^ to virtually screen and computationally design BChE mutants with considerably improved catalytic activity against cocaine, demonstrating that mutations on other amino-acid residues (that are nearby or far away from the active site) could significantly affect the overall hydrogen bonding between the carbonyl oxygen and oxyanion hole and, thus, significantly change the catalytic activity. In light of all these insights, we are currently planning a long-term integrated computational-experimental effort, including extensive virtual screening of the rate-determining transition-state (TS1) structures associated with a large number hypothetical mutants of human BChE and subsequent experimental studies, for rational design and discovery of BChE mutants with improved catalytic activity against ghrelin. A BChE mutant with significantly improved catalytic activity against ghrelin should be therapeutically valuable for use as an exogenous enzyme in obesity treatment.

## Methods

### Construction of the initial BChE-ghrelin binding structures

Starting from an extended backbone conformation of ghrelin, AMBER12 package^43^ was used to model the structure of free ghrelin molecule in water by performing MD simulations for 20 ns. Force-field parameters of the non-standard residues, *i.e. n-*octanoylated Ser3 of ghrelin and caprylic acid, were prepared according to published methods^44^,^45^. X-ray crystal structure of BChE (PDB code: 1P0I, 2.0 Å resolution)^46^ and the simulated structure of ghrelin were used to generate the initial BChE-ghrelin binding complex structure (reactant state RS). Then the simulated system was prepared using a similar protocol described previously^47^. Briefly, AMBER99SB force field was used to model the protein and counter ions (Na^+^)^48^. The TIP3P water model was employed for the solvent^49^. Protonation states of all acidic and basic amino-acid residues were determined by surrounding environment under physiological pH condition (pH 7.4). Hydrogen atoms were added to the PDB file *via* the LEAP module of the AMBER12. The system was solvated initially with TIP3P water for a minimum distance of 10 Å on each side. The N-terminal amino group of ghrelin was manually placed near to the entrance of substrate-binding site for BChE by PyMol^50^. The AMBER12 was used to first guide the N-terminal amino group of ghrelin approaching carboxyl group of Glu197 of BChE, and then guide the carbonyl carbon on the *n-*octanoylated Ser3 side chain of ghrelin to approach the hydroxyl group on Ser198 side chain of BChE. Next, the carbonyl oxygen of the *n-*octanoylated Ser3 of ghrelin was guided to form hydrogen bonds with backbone hydrogen atoms of residues (e.g. Gly116, Gly117, and Ala199) within the oxyanion hole of BChE by using variable distance constraint implemented in the AMBER12 program.

The obtained BChE-ghrelin structure was used as the initial structure for further MD simulations, starting from a 10-ns MD simulation with constrained distances related to Ser3 of ghrelin. The constrained MD simulation was followed by a fully relaxed MD simulation for 20 ns to obtain a stable MD trajectory generating a stable BChE-ghrelin binding structure. The last snapshot of the MD simulation was selected as the initial structure for subsequent Stochastic boundary MD simulation based on the QM/MM method with the QM calculation at the SCC-DFTB level^36^ implemented in the CHARMM program^51^,^52^. Specifically, for the QM/MM calculations, the water molecules located longer than 22 Å away from the hydroxyl oxygen on Ser198 side chain of BChE were deleted from the system. Included in the QM-treated region were the side chains of *n-*octanoylated Ser3 of ghrelin and Ser198, His438, and Glu325 of BChE, and the remaining part of the system was included in the MM-treated region. The divided frontier charge (DIV) link-atom scheme^52^,^53^ was applied to the QM and MM boundary treatment. The SCC-DFTB method with empirical dispersion corrections^54^ implemented in the CHARMM^55^ was used for the QM atoms and the all-hydrogen CHARMM potential function (PARAM27)^37^ was used for the MM atoms. Previous computational studies have demonstrated that the SCC-DFTB based QM/MM calculations on esterase reactions are realiable^56^. The resulting system contained around 6000 atoms, including about 300 water molecules. No non-bonded interaction truncation cut-off was used in the calculations of QM–MM interactions. Within the MM region atoms, the used non-bonded interaction truncation cut-off was 13 Å.

Stochastic boundary MD method^57^ was used with the oxygen atom (O^γ^) of Ser198 side chain as the reference centre. The reaction region was a sphere with a radius (*r*) of 20 Å, and the buffer region extended over 20 Å ≤ *r* ≤ 22 Å. All bonds involving hydrogen atoms in MM region were constrained by using the SHAKE algorithm^58^. The initial structures for the entire stochastic boundary systems were optimized using the steepest descent (SD) and adopted-basis Newton-Raphson (ABNR) methods. A 1-fs time step was used for integration of the equation of motion. The system of reactant complex was gradually heated from 50.0 K to 298.15 K in 100 ps and then 1.5 ns production run was performed for the BChE-ghrelin complex (reactant state RS).

### QM/MM reaction-coordinate calculations

The final RS structure mentioned above was used to perform QM/MM reaction-coordinate calculations at the QM/MM(SCC-DFTB:CHARMM27) level in which the QM calculation was performed at the SCC-DFTB level^36^ while the MM calculation was performed using the CHARMM27 force field^37^ implemented in the CHARMM program. Further, for validation, the QM/MM reaction-coordinate calculations were also carried out at the QM/MM(B3LYP/6-31G*:CHARMM27) level in which the QM calculation was performed at the B3LYP/6-31G* level while the MM calculation was carried out using the CHARMM27 force field. In addition, the geometry optimization at the QM/MM(B3LYP/6-31G*:CHARMM27) level was followed by single-point energy calculation at the QM/MM(B3LYP/6-311++G**:CHARMM27) level, which may be denoted as the QM/MM(B3LYP/6-311++G**:CHARMM27)//QM/MM(B3LYP/6-31G*:CHARMM27) level for convenience. Our previous QM/MM calculations on BChE-involved enzyme reactions^59^,^60^,^61^,^62^,^63^,^64^,^65^,^66^ revealed that these QM levels (B3LYP/6-31G* for geometry optimization and B3LYP/6-311++G** for subsequent single-point energy calculations) are adequate for the QM/MM calculations on these enzymatic reaction systems.

The QM/MM reaction-coordinate calculations were also carried out using the B97-3 functional^38^ or ωM06-D3 functional^39^, instead of the B3LYP functional, at the QM/MM(B97-3/6-31G*:CHARMM27) and QM/MM(ωM06-D3/6-31G*:CHARMM27) levels, followed by the single-point energy calculations at the QM/MM(B97-3/6-311++G**:CHARMM27) or QM/MM(ωM06-D3/6-311++G**:CHARMM27) level. The QM/MM calculations with the B3LYP or B97-3 were carried out by using the GAMESS-US program^67^ interfaced with the CHARMM program^51^,^52^, and the ωM06-D3-based QM/MM calculations were carried out by using the QCHEM program^68^ interfaced with the CHARMM program^69^.

Reaction coordinates (RC’s) used in the QM/MM reaction-coordinate calculations were defined as linear combinations of key internuclear distances involved in the reaction process. The potential energy surface was generated by the adiabatic mapping calculations starting from the last snapshot of QM/MM(SCC-DFTB:CHARMM27) MD simulation on the reactant state (RS) for acylation or acyl-enzyme (AE) for deacylation. The adiabatic mapping calculations at all QM/MM levels were carried out using a uniformed protocol. Specifically, a harmonic constraint with force constant of 10000 kcal mol^−1 ^Å^−2^ was added to the RC to guide the BChE acylation and deacylation reaction processes. The RC value was increased stepwise along the reaction path (from RS to AE for acylation or from AE to PS for deacylation), with a step size of 0.2 Å. An ABNR minimization was carried out under the constraint. The RC value then decreased from the product to the reactant. The forward and backward progresses were repeated until obtaining a smooth and more reliable potential energy surface profile along the reaction path.

In addition, forward and backward intrinsic reaction coordinate (IRC) calculations were also carried out at the QM/MM(SCC-DFTB:CHARMM27) level starting from the optimized TS1 geometry associated with the first-order saddle point on the potential energy surface, in order to make sure that the first-order saddle point associated with TS1 indeed directly connects with the two local minima associated with RS and AE on the potential energy surface.

### Potential mean force (PMF) free energy simulation

The PMF free energy simulation was based on the aforementioned QM/MM(SCC-DFTB:CHARMM27) MD simulations. The umbrella sampling method^70^ implemented in the CHARMM program along with the Weighted Histogram Analysis Method (WHAM)^71^ was used to determine the change of the 2D free energy (PMF) surface as a function of the reaction coordinate. The 2D potential energy maps were first generated for both the acylation and deacylation processes by adiabatic mapping calculations starting from the last snapshot of the QM/MM(SCC-DFTB:CHARMM27) MD simulation on the reaction system. For the 2D free energy maps (including the acylation and deacylation processes), a total of more than four-hundred windows were used and, for each window, 100 ps MD simulation was performed with 50 ps for equilibration. 80ps MD simulation with 50 ps for equilibration was applied for error bar analysis purpose. The force constant of the harmonic biasing potential used in all of the 2D PMF simulations was 150 kcal mol^−1 ^Å^−2^.

## Additional Information

**How to cite this article**: Yao, J. *et al.* Unexpected reaction pathway for butyrylcholinesterase-catalyzed inactivation of “hunger hormone” ghrelin. *Sci. Rep.* **6**, 22322; doi: 10.1038/srep22322 (2016).

## Supplementary Material

Supplementary Information
