# Supplementary material for: Unexpected Reaction Pathway for butyrylcholinesterase-catalyzed inactivation of “hunger hormone” ghrelin
Source: Sci Rep. 2016 Feb 29;6:22322. doi: 10.1038/srep22322 (PMC4770301; doi:10.1038/srep22322)
Supplement: Supplementary Information [file srep22322-s1.pdf]

Supporting Information

Unexpected reaction pathway for butyrylcholinesterase-catalyzed inactivation of "hunger hormone" ghrelin

Jianzhuang Yao,[a,b] Yaxia Yuan,[a,b] Fang Zheng,[a,b] and Chang-Guo Zhan [a,b,]*

[a]Molecular Modeling and Biopharmaceutical Center and [b]Department of Pharmaceutical Sciences, College of Pharmacy, University of Kentucky, 789 South Limestone Street, Lexington, KY 40536

Correspondence to:
Chang-Guo Zhan, Ph.D.
Director, Molecular Modeling and Biopharmaceutical Center (MMBC)
Director, Chemoinformatics and Drug Design Core of CPRI
Endowed College of Pharmacy Professor in Pharmaceutical Sciences
Professor, Department of Pharmaceutical Sciences
College of Pharmacy
University of Kentucky
789 South Limestone Street
Lexington, KY 40536
Phone: 859-323-3943
FAX: 859-257-7585
E-mail: zhan@uky.edu

Supporting Information Available: Additional Figures (S1 to 5) and additional QM/MM reaction-coordinate calculation tests.

Additional Figures

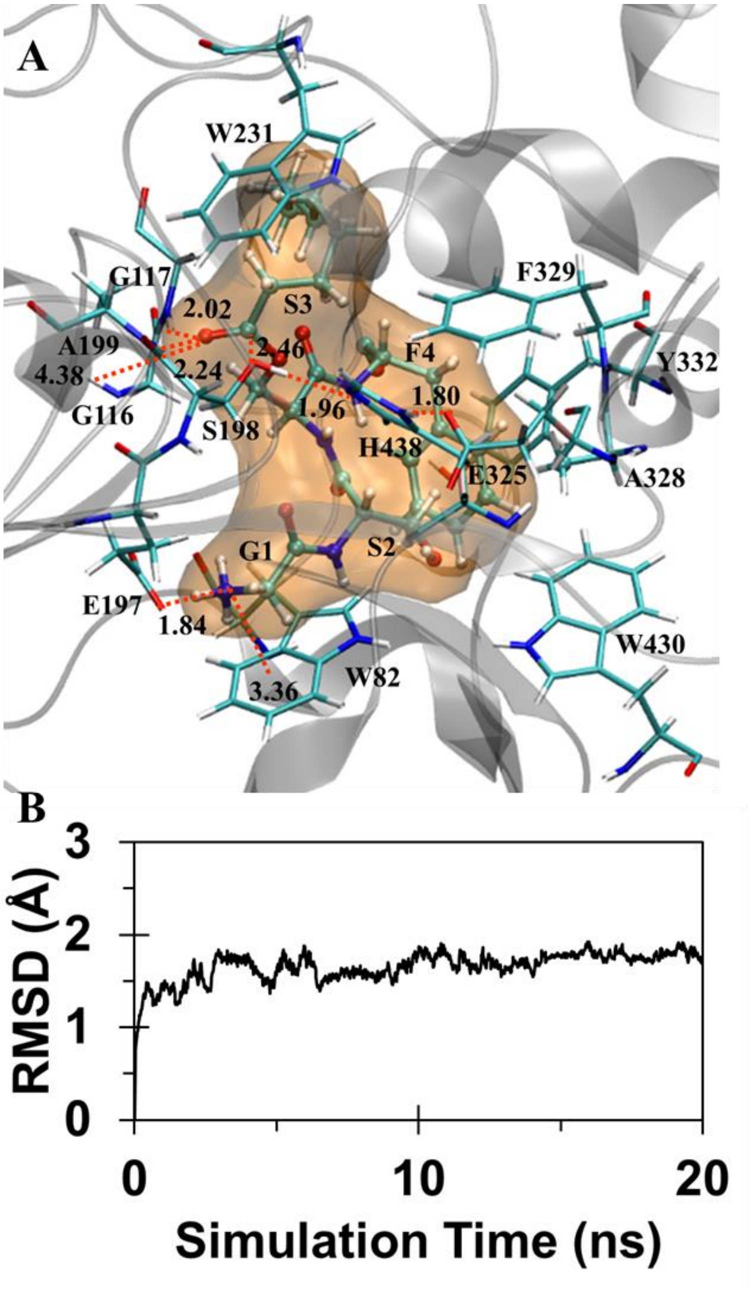

Figure S1. (A) Computationally simulated BChE-ghrelin binding structure. Depicted is the average structure generated by the last 500 ps of the QM/MM(SCC-DFTB:CHARMM27) MD trajectory. The first four residues on the N-terminus of ghrelin are shown in the ball and stick style, the backbone of BChE is shown in the new cartoon style, and key residues of BChE are shown in the stick style. All distances are given in Å. (B) Tracked changes of the positional RMSD values for all atoms of the BChE-ghrelin complex during the 20 ns MD simulation.

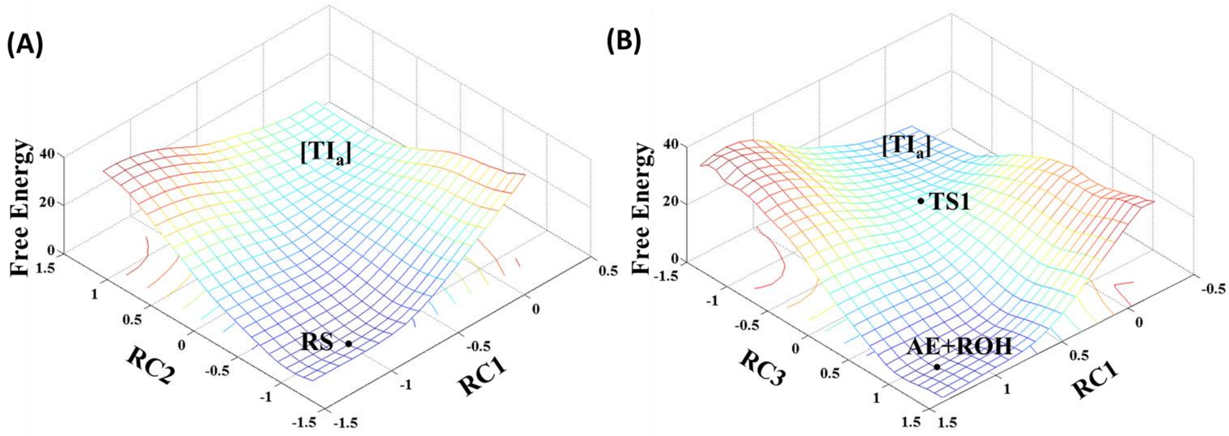

Figure S2. 2D free energy maps obtained from the combined QM/MM(SCC-DFTB:CHARMM27) MD and PMF simulations on the acylation process using two sets of reaction coordinates (RC's): (A) Use of RC1 and RC2 to simulate the formation of the pre-assumed tetrahedral intermediate (TI_a) according to the hypothesized two-step reaction pathway; (B) RC1 and RC3 to simulate the decomposition of the pre-assumed TI_a according to the hypothesized two-step reaction pathway. RC1 = $r(C–O^3) - r(C–O^\gamma)$, RC2 = $r(O^\gamma–H^\gamma) - r(N^\varepsilon–H^\gamma)$, and RC3 = $r(N^\varepsilon–H^\gamma) - r(O^{3}\cdots H^\gamma)$ (see Figures 1 and S4 for the labelled atoms). The energy is in kcal/mol, and the RC's are all in Å. In both panels, [TI_a] indicates the approximate position of the pre-assumed TI_a, although the pre-assumed TI_a does not exist on any of the maps. The maps demonstrate that there is only one transition state (TS1) without an intermediate between RS and AE (or AE + ROH).

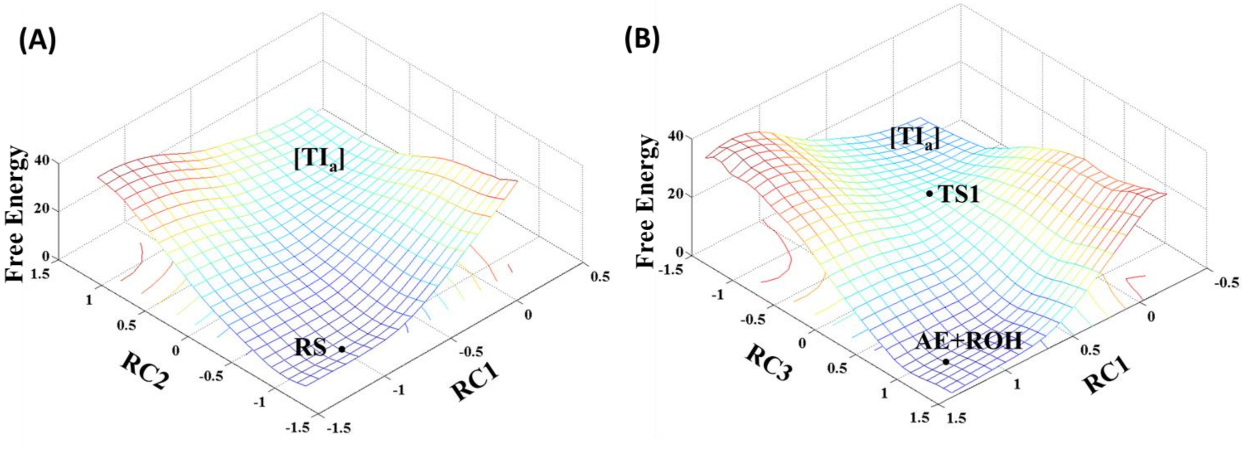

Figure S3. 2D free energy maps obtained from the combined QM/MM(SCC-DFTB:CHARMM27) MD and PMF simulations on the deacylation process using two sets of reaction coordinates (RC's): (A) Use of RC4 and RC6 to simulate the formation of the pre-assumed tetrahedral intermediate (TI_d) according to the hypothesized two-step reaction pathway; (B) RC4 and RC5 to simulate the decomposition of the pre-assumed TI_a according to the hypothesized two-step reaction pathway. RC4 = r(C–O^{γ}) – r(C–O^{w}), RC5 = r(O^{w}–H^{w}) – r(N^{ε}–H^{w}), and RC6 = r(N^{ε}–H^{γ}) – r(O^{γ}–H^{w}). The energy is in kcal/mol, and the RC's are all in Å. The maps demonstrate that the pre-assumed TI_d indeed exists is sandwiched by two transition states (TS2 and 3) between RS and AE + H_2O and PS.

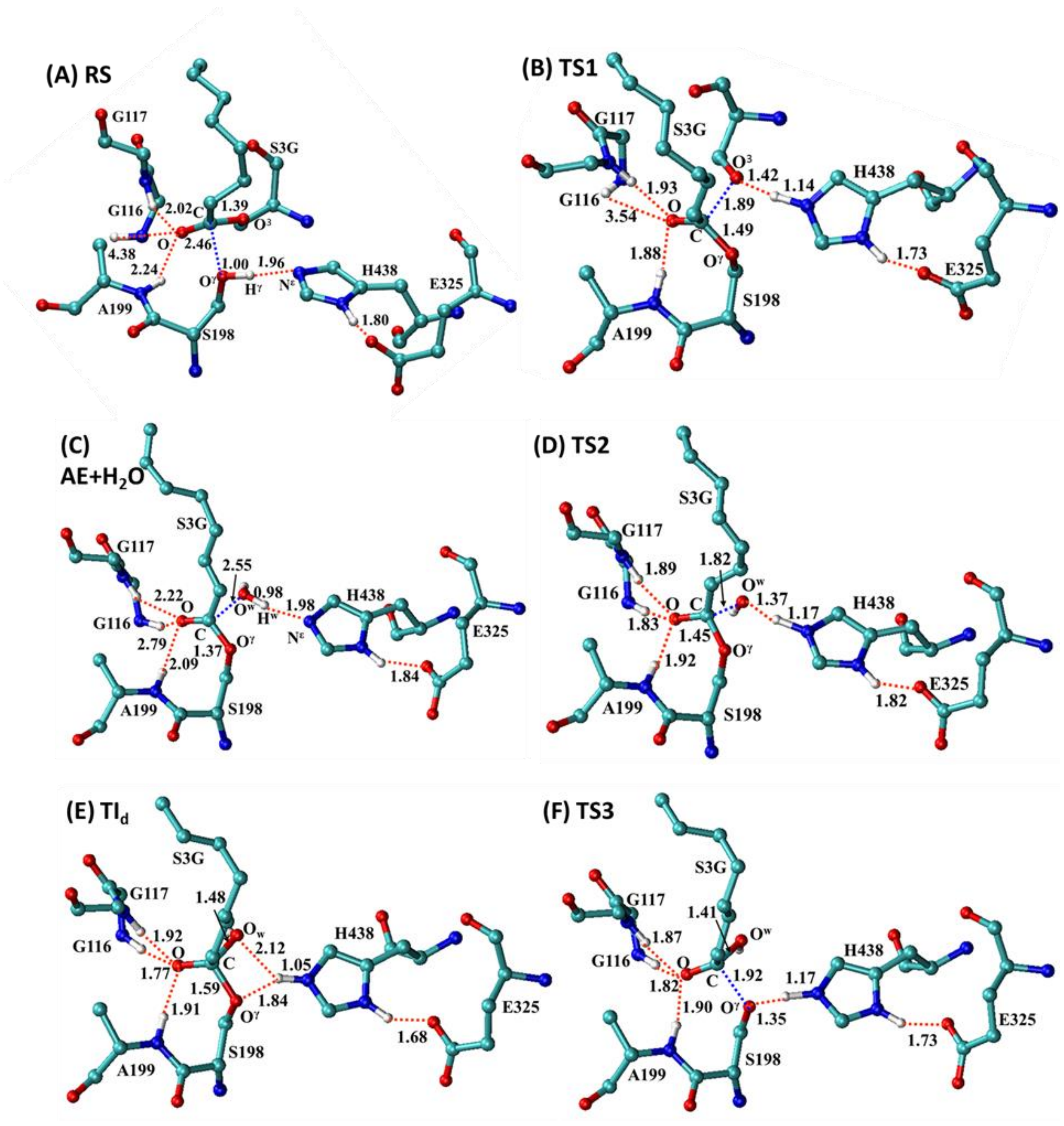

Figure S4. The QM/MM(SCC-DFTB:CHARMM27) MD-simulated average structures of the enzymatic reaction system (BChE-catalyzed hydrolysis of ghrelin) in different states: (A) RS; (B) TS1; (C) AE + H_2O; (D) TS2; (E) TI_d; (F) TS3. For RS, all of the collected snapshots of the last 500 ps (one snapshot for 0.5 ps) of the MD trajectory were used to obtain the average structure. For all other states, all of the collected snapshots of the last 50 ps (one snapshot for 0.5 ps) of the MD trajectory were used to obtain the average structure. The distances are given in Å.

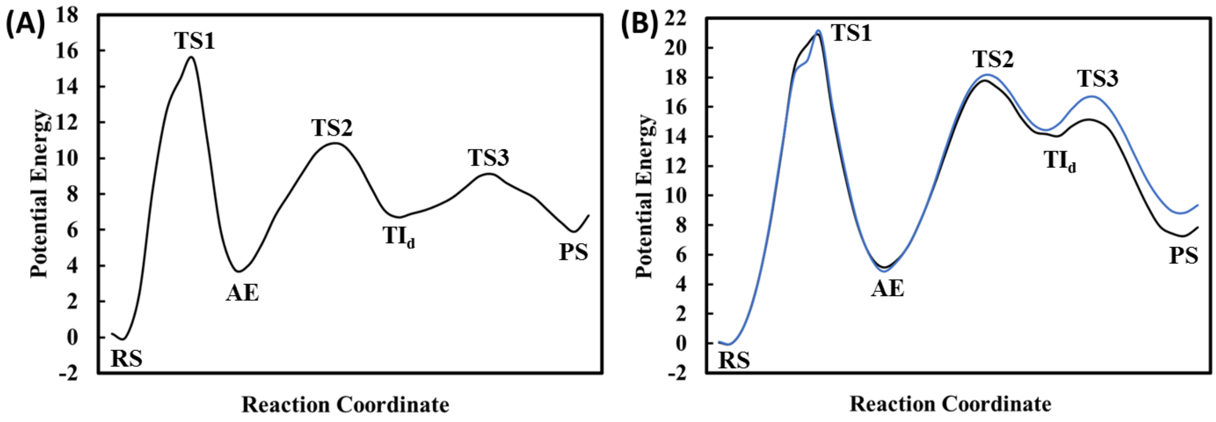

Figure S5. Comparison of the potential energy (kcal/mol) profiles determined by the QM/MM reaction-coordinate calculations on BChE-catalyzed hydrolysis of ghrelin at different levels. (A) The reaction-coordinate calculations at the QM/MM(SCC-DFTB:CHARMM27) level. (B) The reaction-coordinate calculations at the QM/MM(B3LYP/6-31G*:CHARMM27) level (black line); and further single-point energy calculations at the QM/MM(B3LYP/6-311++G**:CHARMM27) level (blue line) using the geometries optimized at the QM/MM(B3LYP/6-31G*:CHARMM27) level.

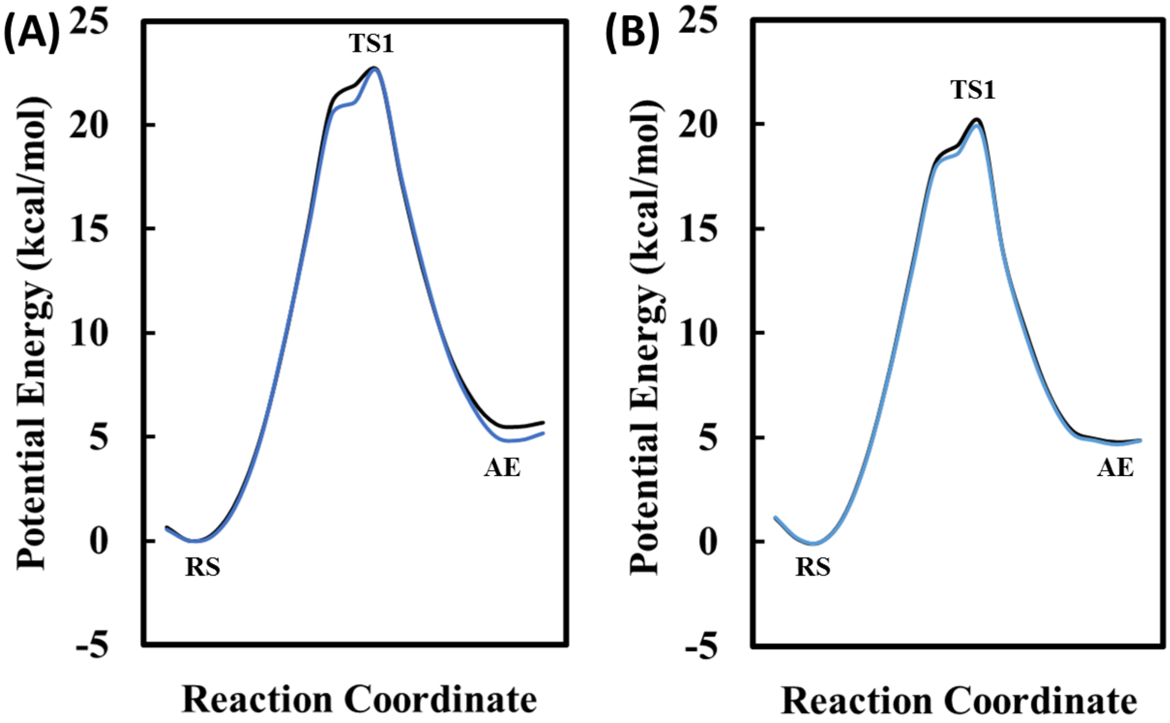

Figure S6. The potential energy surfaces obtained from the QM/MM reaction-coordinate calculations on the acylation process using alternative density functionals instead of B3LYP: (A) Reaction-coordinate calculations at the QM/MM(B97-3/6-31G*:CHARMM27) level (black line), and the single-point energy calculations at the QM/MM(B97-3/6-311++G**:CHARMM27) level (blue line) using the geometries optimized at the QM/MM(B97-3/6-31G*:CHARMM27) level. (B) Reaction-coordinate calculations at the QM/MM(ωM06-D3/6-31G*:CHARMM27) level (black line), and the single-point energy calculations at the QM/MM(ωM06-D3/6-311++G**:CHARMM27) level (blue line) using the geometries optimized at the QM/MM(ωM06-D3/6-31G*:CHARMM27) level.

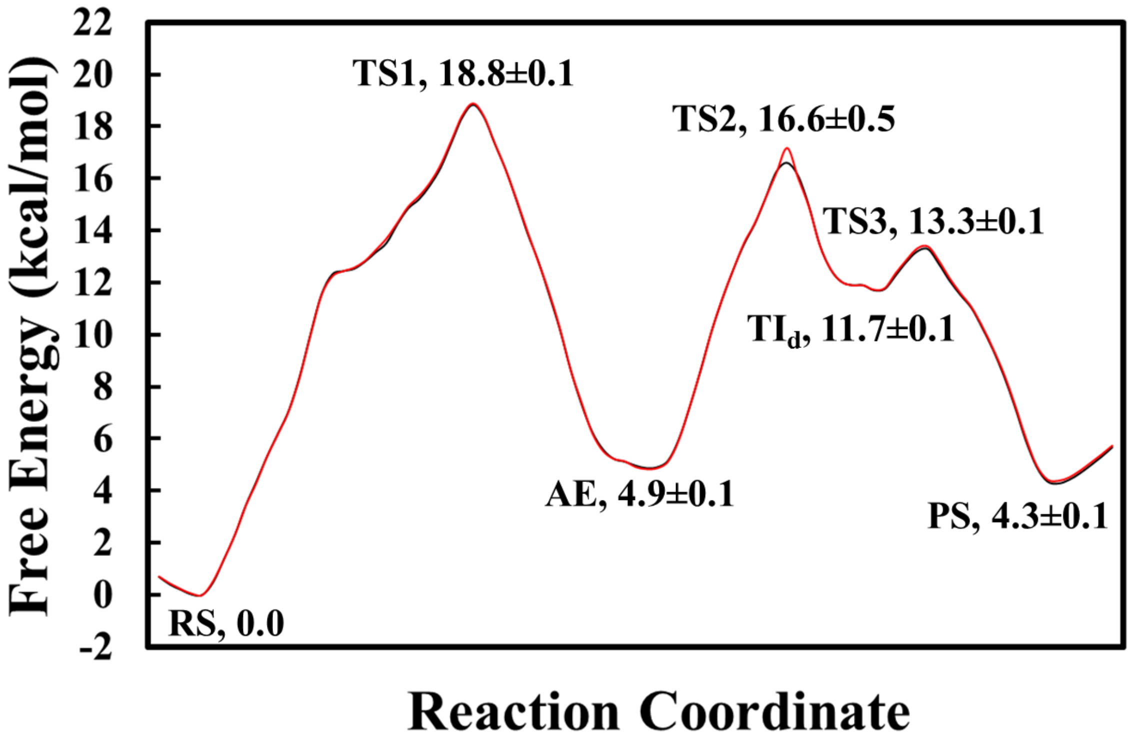

Figure S7. Minimum free energy profiles determined by the QM/MM(SCC-DFTB:CHARMM27) calculations based two-dimensional PMF free energy maps for the entire reaction process (acylation and deacylation) of BChE-catalyzed hydrolysis of ghrelin. The black line (which is the same as that depicted in Figure 4) represents the free energy profile based on the PMF simulations including 50 ps production MD run for each window, and the red line refers to the free energy profile based on the PMF simulations including only 30 ps production MD run for each window. The error bars indicated in the figure are the maximum differences between the calculated free energies associated with the 50 ps production run and the corresponding free energies associated with the 30 ps production run. When the maximum difference is smaller than 0.1 kcal/mol, the error bar is considered as ±0.1 kcal/mol.

Table S1. Absolute QM/MM energies (E in kcal/mol) obtained from the reaction-coordinate calculations at the QM/MM(B3LYP/6-31G*:CHARMM27) level and the single-point energies calculated at the QM/MM(B3LYP/6-311++G**:CHARMM27) level using the geometries optimized at the QM/MM(B3LYP/6-31G*:CHARMM27) level.

State	E(B3LYP/6-31G*:CHARMM27)	E(B3LYP/6-311++G**:CHARMM27)
RS	-730364.32	-730491.04
TS1	-730343.55	-730470.82
AE+ROH	-730359.17	-730486.25
AE+H_2O	-706533.78	-706773.46
TS2	-706521.15	-706760.20
TI_d	-706525.02	-706766.04
TS3	-706524.07	-706763.83
PS	-706531.91	-706771.62

Table S2. Absolute QM/MM energies (E in kcal/mol) obtained from the reaction-coordinate calculations at the QM/MM(B97-3/6-31G*:CHARMM27) level and the single-point energies calculated at the QM/MM(B97-3/6-311++G**:CHARMM27) level using the geometries optimized at the QM/MM(B97-3/6-31G*:CHARMM27) level.

State	E(B97-3/6-31G*:CHARMM27)	E(B97-3/6-311++G**:CHARMM27)
RS	-730594.01	-730902.83
TS1	-730573.95	-730880.29
AE+ROH	-730589.24	-730897.99

Table S3. Absolute QM/MM energies (E in kcal/mol) obtained from the reaction-coordinate calculations at the QM/MM(ωM06-D3/6-31G*:CHARMM27) level and the single-point energies calculated at the QM/MM(ωM06-D3/6-311++G**:CHARMM27) level using the geometries optimized at the QM/MM(ωM06-D3/6-31G*:CHARMM27) level.

State	E(ωM06-D3/6-31G*:CHARMM27)	E(ωM06-D3/6-311++G**:CHARMM27)
RS	-730629.57	-730645.14
TS1	-730607.01	-730625.38
AE+ROH	-730624.08	-730640.47

Additional QM/MM reaction-coordinate calculation tests

Additional QM/MM reaction-coordinate calculations were carried out on the acylation process at the QM/MM(SCC-DFTB:CHARMM27) level using different reaction coordinates (RC's) to further examine the hypothesized two-step reaction pathway. According to the hypothesized two-step reaction pathway, the first reaction step should be formation of tetrahedral intermediate TI_a. In this reaction step, the hydroxyl oxygen (O^{γ}) on Ser198 side chain of BChE would gradually approaches the carbonyl carbon (C) on the *n*-octanoylated Ser3 side chain of ghrelin while the hydroxyl hydrogen (H^{γ}) gradually transfers to the nitrogen (N^{ε}) atom on His438 side chain of BChE and, thus, RC was set as $r(O^{\gamma}–H^{\gamma}) – r(C–O^{\gamma}) – r(N^{\varepsilon}–H^{\gamma})$ for the QM/MM reaction-coordinate calculations on this reaction step. However, according to the QM/MM reaction-coordinate calculations using different RC values, the potential energy always became higher and higher with increasingly larger and larger RC value (data not shown). No saddle point or local minimum has been identified along the RC, suggesting that the hypothesized two-step reaction pathway does not exist for the acylation process of the BChE-catalyzed hydrolysis of ghrelin.
